# Development of a surface enhanced Raman scattering lateral flow immunoassay with prolonged reproducibility and stability over time

**DOI:** 10.1039/d6an00053c

**Published:** 2026-04-02

**Authors:** Sian Sloan-Dennison, Ben Clark, Kathleen M. Scullion, Fiona Smillie, Stacey Laing, Paul Fineran, Joanne Mair, Cicely Rathmell, Dieter Bingemann, Jonathan Faircloth, David Creasey, Neil C. Shand, Chris J. Weir, James W. Dear, Duncan Graham, Karen Faulds

**Affiliations:** a Department of Pure and Applied Chemistry, Technology and Innovation Centre 99 George Street Glasgow UK karen.faulds@strath.ac.uk; b Centre for Cardiovascular Science, The Queen's Medical Research Institute, University of Edinburgh Edinburgh UK; c Wasatch Photonics Morrisville NC USA; d Defence Science and Technology Laboratory, Porton Down Salisbury UK; e Edinburgh Clinical Trials Unit, Usher Institute, University of Edinburgh Edinburgh UK

## Abstract

Lateral flow immunoassays (LFIAs) coupled to surface enhanced Raman scattering (SERS) measurements can produce quantitative outputs with excellent sensitivity. Despite this, they have not been adopted into point-of-care (POC) scenarios, due to the perceived lack of reproducibility and stability in SERS measurements over time. To address this, the performance, stability and reproducibility of a newly developed SERS-LFIA designed for the detection of liver injury has been assessed to investigate if the tests could yield the same results six-months after preparation. The SERS-LFIA incorporated silica coated Raman active gold nanoparticles to increase the stability when used with human serum samples and the SERS signals of the test and control lines were analysed using a handheld Raman reader to mimic its use at the POC. The relative standard deviations of key metrics, including reproducibility and variation in signal were assessed over the course of a 6 month stability study using a range of methods that aimed to standardise the output. Test line intensity, test/control ratio and slope linear regression outputs were all used to assess the SERS-LFIA results with the best reproducibility being achieved when normalising the test *via* the slope linear regression method. After 5 months the output only decreased by 11% for a sample spiked with a clinically relevant concentration of liver injury biomarker, demonstrating the test was still performing as intended. Overall, we have shown that SERS-LFIA can produce similar outputs after 5 months of storage at room temperature demonstrating their stability and robustness.

## Abstract 2

Lateral flow immunoassays (LFIAs) coupled to surface enhanced Raman scattering (SERS) measurements can produce quantitative outputs with excellent reproducbility over a prolonged period of time.

## Graphical abstract

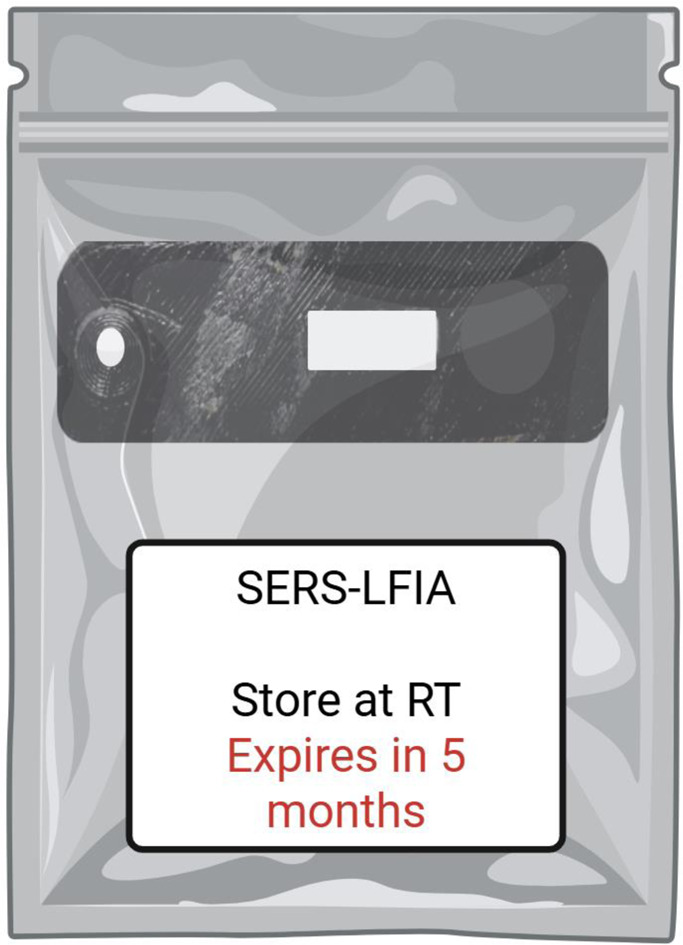

## Introduction

Surface enhanced Raman scattering-lateral flow immunoassays (SERS-LFIAs) are an emerging platform that combine the point-of-care (POC) nature of LFIA tests with sensitive and quantitative SERS measurements.^1^ To achieve this, SERS-LFIAs use conjugates consisting of nanoparticles (NPs) functionalised with a Raman reporter and recognition motif, most commonly a detection antibody, to form a sandwich immunoassay with a biomarker present in the sample and capture antibody on the test line. The test line can then be interrogated by an appropriate laser excitation wavelength and the intensity of the SERS signal of the Raman reporter is used to determine the concentration of the biomarker present in the sample.^2^

Recently, SERS measurements have been transferred from large Raman microscope systems, where the test line is mapped taking up to 20 minutes per sample, to portable Raman spectrometers that can analyse the test line in seconds. SERS-LFIAs paired with portable Raman spectrometers have been used to detect many clinically relevant biomarkers, producing results that are as sensitive as those achieved in larger systems but in much faster time frames.^3–5^ They also require less space and are more user friendly, all of which are desirable characteristics for POC platforms.^6,7^ The potential of the platform has been shown by Joung *et al.* who investigated the performance of a portable SERS-LFIA reader for on-site testing of clinical nasal mucosa samples to diagnose SARS-CoV-2.^8^ The authors demonstrated that upon testing 54 clinical samples, the SERS-LFIA had a sensitivity of 96%, a considerable improvement on the visual analysis, which had a sensitivity of only 57%. Xiao *et al.* used a portable SERS-LFIA reader for the multiplexed detection of cancer biomarkers alpha-fetoprotein, carcinoembryonic antigen and prostate-specific antigen in clinical serum samples with a detection success rate of 100%.^4^ Recently, we developed a SERS-LFIA paired with a portable Raman spectrometer, also known as a handheld Raman reader (HRR), to detect the drug induced liver injury (DILI) biomarker cytokeratin-18 (K18). The test achieved diagnostic accuracy for DILI with a specificity of 94% and sensitivity of 82% in 199 clinical samples, indicating the strength of SERS-LFIA to rapidly identify patients as healthy or unhealthy, allowing for individualised treatment pathways.^9^ There are many benefits of SERS-LFIAs, including high sensitivity, quantitation, rapid time to result and minimal sample preparation. Overall, this can lead to an improvement in patient stratification, removing the need for lab-based tests when quick determination is needed.

Although positive results have been achieved using SERS-LFIAs, they have not yet been readily adopted in clinical practice. This is mainly due to the common misconception that SERS measurements offer inadequate reproducibility and stability over time. However, there are many methods, including the incorporation of internal standards, normalisation methods, and careful probe design, that have been used to overcome this and can be incorporated into SERS-LFIA.^10^ For example, internal standards have been incorporated into a SERS-LFIA by Peng *et al.* whose design included graphene oxide/gold NP embedded onto the nitrocellulose membrane, which acted as the internal standard.^3^ The SERS intensity of the graphene oxide, which was always present in the SERS signal, was used to calibrate the Raman intensity fluctuations of the molecules caused by uneven distribution of hotspots, demonstrating a 1.65-fold improvement in reproducibility. Inspired by LFIA image analysis methods, widely used in ovulation urine tests where dedicated apps or imaging software generate a test-to-control pixel intensity ratio to normalise assay output, the SERS-LFIA signal can similarly be normalised to improve reproducibility. Among these approaches, the test-to-control intensity ratio is the most commonly applied method for compensating for test variability in lateral flow assays.^11,12^ For example, the output of a SERS-LFIA detecting SARS-CoV-2 from nasopharyngeal swabs has been normalised using test-to-control line SERS intensity and although no comparison was made to using the test line intensity alone, a sensitivity of 96% was achieved when run with human samples.^8^

Although internal standards and normalisation can improve the reproducibility, it is also important to ensure the long-term stability of the SERS signal, which is often linked to the NPs themselves, due to their susceptibility to aggregation, and the stability of the Raman reporter which can be prone to degradation or desorption from the metal surface. Both will affect the intensity of the SERS signal over time. To avoid instability, the NPs and the Raman reporter should be sufficiently protected. Bovine serum albumin (BSA) is readily used to protect conjugates from aggregation due to its easy attachment and good biocompatibility. Xiao *et al.* demonstrated the reproducibility of SERS-LFIAs, which used BSA protected conjugates, to detect three different antigens with relative standard deviation (RSD), also termed coefficient of variation, values of 13, 2.4 and 5% based on the intensity of one peak in the SERS spectrum compared across 8 different LFIA strips.^4^ This was a favourable result given for most bioanalytical methods, an RSD 10–15% is typically considered acceptable.^13^ BSA has also been used to protect gold nanorods, and when applied in SERS-LFIAs, an RSD of 4.7% was achieved using serum samples.^14^ RSD values for 5 independent SERS-LFIA tests for the SARS-CoV-2 S protein antibody at various concentrations (10, 1 and 0.1 ng mL^−1^) were calculated to be 6.7, 4.2 and 12.4%, respectively, again suggesting that SERS-LFIAs can be highly reproducible.^15^ In this example, a core silica nanoparticle was used as the template onto which a plasmonic silver layer was grown, resulting in a stable, monodispersed conjugate with BSA offering additional protection to the silver NP surface. Silica can also be used to encapsulate a core plasmonic NP, essentially blocking the NP surface from the sample environment that could result in aggregation and instability. Jeon *et al.* investigated the thermal stability of a SERS-LFIA that used silica encapsulated gold NPs and demonstrated that good reproducibility and stability was achieved after they were stored at high temperatures (45 °C) for 24 hours.^16^ Silica encapsulation of gold NPs has also been used in a SERS-LFIA to detect C-reactive protein and serum amyloid A.^17^ The authors of this publication demonstrated that using silica in the production of SERS nanotags resulted in a very low RSD between different batches (4.6%), and after 50 days of storage, their SERS signal exceeded 70% when compared to that of newly prepared SERS nanotags. Silica coated NPs can be readily dispersed onto the conjugate pad of the SERS-LFIA using additives such as Tween 20 and BSA, followed by freeze drying the pad in a vacuum. When this method was applied during the preparation of a SERS-LFIA, the performance of the test after 60 days of storage did not show any obvious reduction in the SERS signal of the test line.^15^

The decrease in SERS-LFIA performance is not exclusively linked to conjugate stability. The degradation of the antibodies on the NPs and test/control lines will also affect the formation of the sandwich complex in the immunoassay.^18^ Various studies have demonstrated that this is linked to storage conditions, such as temperature and humidity, and can be overcome by storing the LFIA strips in sealed foil bags with a desiccant (silica) to limit exposure to moisture.^19^ Another way to improve stability is to store SERS-LFIA strips in a zip-lock bag and place them in a refrigerated dryer at 4 °C. Jiang *et al.* demonstrated that when this storage condition was used, their SERS-LFIA had the same performance over a 6 month period.^5^ However, if SERS-LFIAs are to be used in a POC setting, storing using these conditions would require extra equipment. This may not always be possible due to space and power restrictions in POC settings and could also prove costly due to additional running and maintenance costs. Ideally, the SERS-LFIA strip should be able to be stored at room temperature until use, with no additional measures.

Here, we have investigated the stability and reproducibility of our recently developed SERS-LFIA test that was designed for the detection of the DILI biomarker K18 using a HRR. The SERS-LFIA used silica-coated nanoparticle conjugates and was stored in foil pouches containing silica desiccant at room temperature. Test performance was assessed at multiple time points: Day 0, 2 weeks, and 1, 2, 3, 5, and 6 months post-preparation to assess the stability of the test over time. At each time point, fresh serum samples spiked with clinically relevant concentrations of K18 were applied to the strip. 0 and 25 ng mL^−1^ concentrations represented healthy status and 100 ng mL^−1^ representing a patient with DILI. The SERS signal was measured using the HRR equipped with line illumination, optimised for POC clinical use to deliver rapid and reproducible results. The SERS signal intensities from both the test and control lines were recorded. Data was analysed using three different output methods: absolute test line intensity, the test-to-control (T/C) signal ratio, and the slope derived from linear regression analysis of control *versus* test line intensity.

## Experimental

### Materials

Sodium tetrachloroaurate(iii) dihydrate (NaAuCl_4_–H_2_O), sodium citrate, 4,4′-dipyridyl (DIPY), 3-(aminopropyl) trimethoxysilane (APTMS), sodium silicate, boric acid, borate, sodium chloride, Tween 20 and sucrose were all purchased from Merck (UK). PBS tablets were purchased from Oxoid. Recombinant human cytokeratin 18 protein, recombinant anti-cytokeratin 18 antibody (capture and detection) and goat anti-rabbit IgG were all purchased from Abcam (USA). Whatman MF1 bound glass fibre filter (conjugate pad), Whatman standard 14 (sample pad), Whatman CF6 dipstick pad (absorbent pad) and nitrocellulose membrane FF170HP were purchased from Cytiva (USA). Polylactic acid (PLA) 3D printer filaments were purchased from Farnell (UK). Whole blood was collected, in serum gel tubes, from healthy volunteers following informed consent and ethical approval (21-EMREC-041) at The University of Edinburgh. It was stored at −80 °C.

### Equipment

Gold nanoparticles (Au NPs) were characterised using an Agilent Cary 60 UV-Vis spectrophotometer, Malvern Zetasizer Nano ZS and CBEx handheld Raman spectrometer with 785 nm laser excitation. Transmission electron microscopy (TEM) imaging was carried out at the University of Glasgow using a JEOL JEM-1400Flash at an 80 kV accelerating voltage. Samples were prepared by drop-casting 1 μL of sample onto 200 mesh carbon-coated copper TEM grids. Antibody test and control lines were added using an Imagene Technology lateral flow reagent dispenser. A BioDot CM500 Guillotine was used to cut nitrocellulose sheets into 5 mm strips that were laminated using a Crenova Laminator. The PLA cassettes were 3D printed using an Ultimaker S5. The test and control lines were measured using a bespoke Wasatch Photonics handheld Raman reader (HRR) with 785 nm laser excitation.

### Gold nanoparticle synthesis

All glassware was cleaned using aqua regia (3 parts hydrochloric acid to 1 part nitric acid) and rinsed with water prior to use. Au NPs were synthesised using a refined Turkevich synthesis^20,21^ by adding NaAuCl_4_–H_2_O (67.5 mg) to distilled water (500 mL) in a 1 L, 3 necked round bottom flask. The solution was heated to 98 °C for 30 minutes followed by the addition of sodium citrate (60.5 mg) and heated for an additional 15 minutes before being cooled to room temperature. Constant stirring was maintained throughout using a glass linked stirrer.

### Raman reporter and silica encapsulation

Au NPs (100 mL) were added to a 250 mL conical flask with a stirrer bar inside. DIPY (300 µL, 100 µM) was added to the solution which was stirred for 5 minutes. APTMS (150 µL, 3 mM) and sodium silicate (SiO_2_, 1.5 mL) were then added. The solution was heated to 98 °C with constant stirring for 30 minutes and the resulting Au-DIPY–SiO_2_ NPs were left to cool whilst being stirred overnight. Au-DIPY NPs were prepared by adding Au NPs (10 mL) to DIPY (30 µL, 100 µM).

### Nanoparticle stability study

Samples containing either 1 mL of Au-DIPY NPs or Au-DIPY–SiO_2_ NPs were incubated with 0, 5, 10 or 15 µL of healthy human serum. After 1 hour, the samples were characterised using extinction spectroscopy, dynamic light scattering (DLS) and SERS using a CBEx spectrometer with 785 nm laser excitation.

### Conjugate functionalisation

Borate buffer (10 mM, pH 9, 100 µL) was added to Au-DIPY–SiO_2_ NPs (1 mL) in a low-binding microtube. Recombinant anti-cytokeratin 18 antibody (AB, 2 µL, 1 mg mL^−1^) was added to the solution and left to shake for 2 hours. Bovine serum albumin (BSA, 100 µL, 1% solution) was then added, and the solution left to shake for an additional 30 minutes. The resulting Au-DIPY–SiO_2_-AB NPs were characterised using extinction spectroscopy, DLS and SERS using a CBEx spectrometer with a 785 nm excitation. The Au-DIPY–SiO_2_-AB NPs were then centrifuged at 2000 relative centrifugal force (RCF) for 20 minutes, the supernatant removed, and the pellet resuspended in 100 µL of double distilled deionised water. They were then stored at 5 °C until required.

### SERS-LFIA assembly

Capture recombinant anti-cytokeratin 18 antibody (1 mg mL^−1^) and goat anti-rabbit IgG (1 mg mL^−1^) were added to the nitrocellulose section of the lateral flow sheet using an Imagene Technology lateral flow reagent dispenser with a 300 mm dispense distance, 30 µL mm^−1^ aspirate rate, 0.1 µL mm^−1^ dispense rate and 15 mm s^−1^ speed. The sheets were left to dry in an oven overnight (37 °C, 16–24 hours).

Sample pad 1 (30 cm × 1 cm) was treated with NaCl (1% in distilled water), sample pad 2 (30 cm × 1.5 cm) was treated with Tween 20 (5% in distilled water) and the conjugate pad (30 cm × 1 cm) was treated with sucrose and Tween 20 (1% and 0.5% in distilled water). The pads were then dried in an oven (37 °C, 3 hours). The treated conjugate pads were then cut into 5 mm strips, and the concentrated Au-DIPY–SiO_2_-AB NPs solution (8 µL) was pipetted onto the pad. The pads were then dried in the oven (37 °C, 3 hours).

To assemble the LFIA, tape 1 on the antibody-treated nitrocellulose plastic backed sheet was removed first. The 30 cm × 2 cm absorbent pad was added to this section. Tapes 2 and tape 3 were then removed. Sample pad 2 was added 0.5 cm from the bottom of the strip. Sample pad 1 was added to the bottom of the strip with an overlap between pads 1 and 2. The sheet was laminated and left overnight to ensure adhesion between the pads and sheet. The sheet was then cut into 5 mm strips using a BioDot guillotine. The 5 mm conjugate pad loaded with Au-DIPY–SiO_2_-Ab NPs was added underneath the 2nd sample pad and the strip was laminated again.

The cassettes that hold the lateral flow strip were 3D printed on an Ultimaker S5 with a 0.4 mm extrusion point using black tough PLA filament. The strip was then positioned into the 3D-printed cassette. The devices were placed in a sealed foil packet containing a silica sachet and stored in darkness at room temperature (20 °C).

### Calibration study

Calibration samples containing 25 µL of healthy human serum were added to 75 µL of lateral flow buffer (10 mM PBS, 0.5% Tween 20) and spiked with 0, 10, 25, 50, 100 or 200 ng mL^−1^ of K18. This was prepared in triplicate using three different healthy serum samples for each calibration set. 100 µL of each sample was then applied to the sample port of the SERS-LFIA and left to run for 20 minutes. The SERS signal of the test and control lines were measured using a Wasatch Photonics HRR with 785 nm laser excitation, 3.6 mW laser power and 1 second acquisition.

### Long term stability study of the SERS-LFIA

On the day of preparation (day 0), 9 SERS-LFIAs were removed from the foil packet and tested. For each SERS-LFIA, samples containing 25 µL of healthy human serum were added to 75 µL of lateral flow buffer and spiked with 0, 25 or 100 ng mL^−1^ of K18. Serum from the same donor collected at one time point was used throughout the time study. To avoid freeze thaw, 25 µL of serum was aliquoted into Eppendorf tubes and thawed prior to use. Each concentration was prepared in triplicate. 100 µL of each sample was then applied to the sample port of the SERS-LFIA and left to run for 20 minutes. The SERS signal of the test and control lines were measured using a Wasatch Photonics HRR with 785 nm laser excitation, 3.6 mW laser power and 1 second acquisition. A photograph of the SERS-LFIA was captured using an iPhone 14 plus after 20 minutes. SERS-LFIA tests were stored for 2 weeks, 1 month, 2 month, 3 month, 5 month, and 6 months and then tested using the same procedure as day 0.

### Data analysis

SERS spectra obtained from Enlighten software was baseline corrected using Matlab software. The peak intensity of the 1230 cm^−1^ peak was obtained for each spectrum. To calculate the SERS ratio of a SERS-LFIA, the intensity of the 1230 cm^−1^ peak obtained from the test line was divided by the intensity of the 1230 cm^−1^ peak obtained from the control line. To obtain the SERS slope *via* linear regression analysis, the intensity of the control spectrum was plotted against the intensity of the test spectrum. A linear line of best fit was applied to the data. The equation of the line was found and the gradient used as the SERS slope value that was used for comparison.

## Results and discussion

**Fig. 1:**
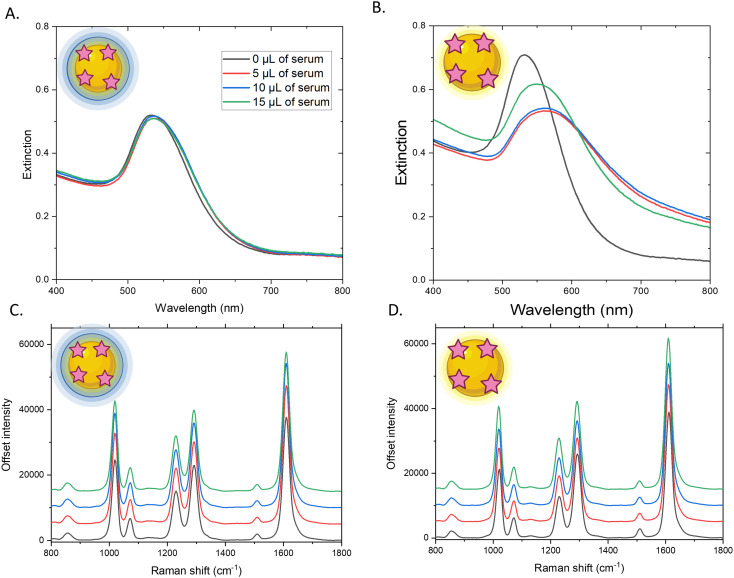
Extinction and offset SERS spectra of Au-DIPY–SiO_2_ NPs (A and C) and Au-DIPY NPs (B and D) 1 hour after being incubated with 0 µL (black), 5 µL (red), 10 µL (blue) and 15 µL (green) of healthy human serum. Solution SERS spectra were collected with a 785 nm laser excitation, 80 mW laser power, 1 second acquisition. SERS Spectra were offset for clarity.

To investigate the stability and reproducibility in performance of a POC SERS-LFIA platform over time, the stability of the SERS active conjugates were initially investigated. This was carried out by synthesising citrate capped Au NPs that were then functionalised with the Raman reporter DIPY and encapsulated in a silica shell. DIPY was selected as it provides a strong SERS signal, without the need for NP aggregation. This synthesis has been well established by the group and creates a thin silica shell surrounding the Au NPs.^22,23^ The resulting Au NPs coated in silica and DIPY (Au-DIPY–SiO_2_ NPs) were characterised using dynamic light scattering, shown in Table S1, which indicated a core Au NP of 56 nm surrounded by a thin silica shell of around 6 nm thickness. We also compared the SERS signal from Au-DIPY NPs (not coated in silica) and the Au-DIPY–SiO_2_ NPs at similar optical densities and demonstrate that coating the Au NPs with a silica shell does not cause aggregation or dampen their SERS performance, Fig. S1. Strong SERS signals from DIPY are still clearly observed after shell formation, indicating that the silica layer does not hinder signal enhancement. This preservation of signal intensity is important and indicates that robust SERS signals will therefore be obtained from the test and control line in the assay. As reported in the literature, the silica shell provides additional stability when the conjugates are exposed to complex sample matrices, including serum, by reducing the interaction between the NP surface and proteins in the matrix that could induce aggregation and/or displace the Raman reporter on the NP surface, resulting in poor SERS-LFIA performance.^24^ To investigate the stability of the silica shell, the Au-DIPY–SiO_2_ NPs were incubated with different volumes of healthy human serum (5, 10 or 15 µL) and the change in extinction, size, zeta potential and SERS signals were characterised and compared to the results of the same experiments carried out on Au-DIPY NPs without any protective silica shell. This is shown in Fig. 1 and Table S1.

As shown in Fig. 1A, the addition of 5, 10 and 15 µL of healthy serum did not induce aggregation in the Au-DIPY–SiO_2_ NPs but did aggregate the Au-DIPY NPs, observed by the peak broadening and dampening in Fig. 1B. This demonstrated that the silica shell had protected the Au NPs from adverse interactions with proteins in the serum. The size and zeta potential measurements also confirmed aggregation of the Au NPs, with very large (56 nm to 1836–2869 nm) and positively charged (−34 mV to 0.1–62 mV) NPs being produced after Au-DIPY NPs were incubated with serum, shown in Table S1. This was likely due to proteins within the serum binding to the negative Au NP surface, replacing some of the negatively charged citrate molecules on the Au NPs resulting in an increase in charge and aggregation. When the Au-DIPY–SiO_2_ NPs were incubated with serum, the silica coated NPs also increased slightly in size from 68 nm to 90–100 nm and became more positive (−23 mV to −10 mW). This indicates that proteins in the serum still interact with the silica surface, increasing their size and surface charge, however the core Au NP remained protected as evidenced by the fact there was no change in the extinction spectrum (Fig. 1A) or large increase in size. Interestingly, the serum addition did not cause significant changes in the SERS signal for either sample, Au-DIPY–SiO_2_ NPs (Fig. 1C) and Au-DIPY NPs (Fig. 1D), with only a slight increase in the SERS signal being observed for each serum volume. With the large amount of aggregation that occurred when the serum was added to Au-DIPY NPs, it was expected that the SERS signal would show an increase, however the data suggests that the protein coating may prevent the surface of the NPs being close enough for their plasmons to couple, which would have led to an increase in SERS signal. Despite the SERS signal being unaffected by the addition of serum, without the protection of silica the Au NPs aggregated, which would have a detrimental effect on their flow and performance in the SERS-LFIA.

To use Au-DIPY–SiO_2_ NPs in the SERS-LFIA, they were functionalised with K18 detection antibodies (AB) and bovine serum albumin (BSA). The conjugates, Au-DIPY–SiO_2_-AB NPs, were then again characterised using extinction spectroscopy, dynamic light scattering and by SERS using 785 nm laser excitation. The results shown in Fig. S2 indicate that they are stable and suitable for use in the SERS-LFIA. To assess the stability of the resulting conjugates when used in a SERS LFIA, 63 SERS-LFIAs devices were assembled, placed in foil packets with a silica desiccant pack, and stored at room temperature in a dark cupboard. The SERS-LFIA performance was then tested over the course of six months.

**Fig. 2:**
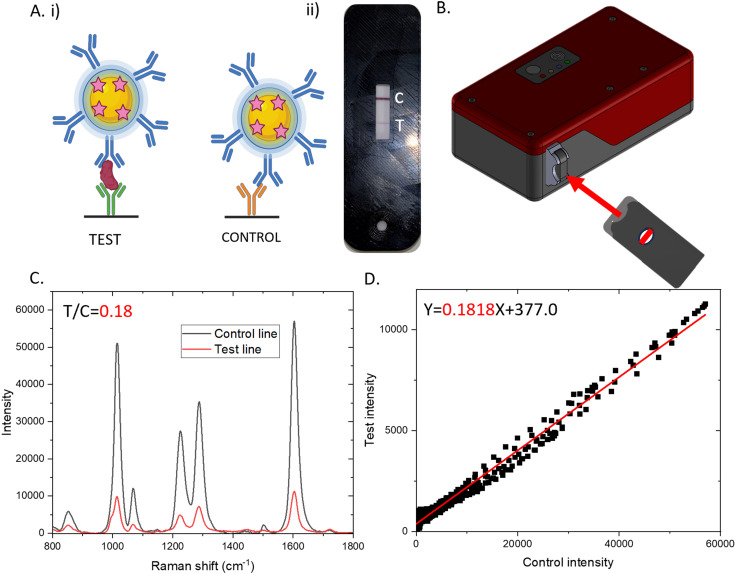
A. (i) Schematic of sandwich complex that forms between Au-DIPY–SiO_2_-Ab NP conjugate, K18 and detection AB (test line) and Au-DIPY–SiO_2_-AB NP conjugate and control AB (control line). (ii) Result of LFIA run with a serum sample spiked with 100 ng mL^−1^ of K18. B. Handheld Raman reader used to analyse LFIA. The LFIA is placed into cassette and inserted into the slot in the HRR. C. SERS spectra obtained from test (red) and control (black) line from LFIA shown in A (ii). The SERS signal of the test and control lines were measured using a Wasatch Photonics HRR with 785 nm laser excitation, 3.6 mW laser power and 1 second acquisition. T/C ratio obtained by finding the intensity of the 1230 cm^−1^ peak in the test and dividing it by the 1230 cm^−1^ peak intensity of the control. D. Linear regression analysis of LFIA shown in A with slope obtained by plotting the intensity of the control spectrum against the intensity of the test spectrum. The equation of the line is shown with the gradient (slope) output shown in red.

The stability and performance of the SERS-LFIAs were assessed over time by testing in triplicate at multiple time points following preparation (Day 0, 2 weeks, and at 1, 2, 3, 5, and 6 months). Each test employed serum samples from the same donor, spiked with 0, 25, or 100 ng mL^−1^ of K18 protein. In the presence of K18, a sandwich complex is formed on the nitrocellulose strip between the Au-DIPY–SiO_2_-AB NPs, the target K18 protein, and the immobilised detection antibody. This interaction produces a red/purple test line, indicating the presence of K18. A control line consistently appears, as the antibody on the Au-DIPY–SiO_2_-AB NPs binds to the antibody immobilised at the control line, as illustrated in Fig. 2A.

20 minutes after sample addition, the SERS signal from both the test and control lines were measured using the HRR shown in Fig. 2B. The HRR was specifically designed for POC settings, such as emergency departments, as it is compact, lightweight and operates at low laser power for safe use. It employs line illumination, in contrast to conventional spot interrogation, allowing the entire line to be measured in a single acquisition. This reduced the analysis time and eliminated the need for multiple measurements per line. To further streamline the process, custom test and control inserts were used to position the SERS-LFIA cassette to allow only the relevant line (test or control) to be visible through a viewing window. This is shown in Fig. 2B. This ensures accurate laser alignment with the intended measurement area, reducing user error and simplifying operation, a key advantage for use at the POC. An example of the SERS spectra from both the test and control lines taken from a SERS-LFIA run with serum spiked with 100 ng mL^−1^ of K18 are shown in Fig. 2C.

As shown in Fig. 2C, the characteristic signal of the Raman reporter molecule, DIPY, is evident in both spectra. However, as expected, the signal is significantly more intense on the control line due to a higher accumulation of NPs, which are subsequently probed by the laser. The output of the LFIA can be quantified in three distinct ways. (1) Test line intensity: this method involves determining the absolute intensity by measuring the peak height of a selected Raman reporter peak in the test line spectrum. (2) Test-to-control ratio (T/C): this approach calculates the ratio of the Raman reporter peak height in the test line to the corresponding peak height in the control line. (3) Slope: here, linear regression is applied to assess the relationship between the SERS spectra of the control and test lines. Conventionally, the reproducibility of SERS-LFIA is assessed by comparing the absolute intensity of a characteristic peak in the SERS spectrum at the test line. While this approach provides a direct measure of signal consistency, it does not account for variability introduced by changes in the conjugates or differences in the quantity of conjugate dispensed on each strip. To address this, the ratio and slope outputs help correct for potential signal changes caused by factors such as degradation of the SERS activity of the conjugate overtime, which could underestimate K18 concentration. In the representative example shown in Fig. 2C, the measured intensity of the 1230 cm^−1^ peak is 4559, the T/C ratio is 0.17, and the slope derived from linear regression is 0.1824 (as calculated in Fig. 2D). The 1230 cm^−1^ peak was selected for test line intensity and for T/C ratio to avoid any background Raman signal from the plastic backing of the LFIA strip. An image of the SERS-LFIA, corresponding test and control line SERS spectra, ratio and slope from tests run with serum spiked with 0, 25 and 100 ng mL^−1^ on Day 0 are presented in Fig. S3. As expected, the intensity, ratio and slope values increase as the K18 concentration does and we can clearly differentiate between the three concentrations.

This normalisation approach can also offer robustness against variability introduced by the complex and heterogeneous nature of human serum. Serum composition varies between individuals due to physiological and lifestyle factors, including age, sex, genetic background, diet, physical activity, and health status, which can influence nonspecific protein interactions with the NP surface. Such interactions may reduce the availability of capture antibodies for analyte binding at the test line. However, because the control line also depends on the same Au–Ab NP conjugate, these matrix effects are reflected in both lines and are thus mitigated by normalisation. Therefore, this allows for improved comparability of results across different patient samples and over extended storage durations. To investigate the effect of different serum matrices on test performance and to demonstrate the value of signal normalisation, a K18 calibration study was performed on the SERS-LFIA in triplicate using healthy human serum obtained from three different donors. The tests were analysed using the HRR, and the test line intensity, test-to-control ratio, and slope outputs at each concentration were obtained (Fig. S4A–C). As shown in Fig. S4A, sera 2 and 3 produced similar test line intensities across the concentration range. In contrast, serum 1 generated substantially higher SERS intensities, particularly at higher concentrations. While this could initially suggest assay instability, the uniformly elevated response observed for serum 1 instead indicates that differences in serum composition likely enhanced binding at the test line. This would result in a greater accumulation of NPs, and consequently a higher SERS signal. Importantly, this matrix-dependent effect can be effectively mitigated by normalising the signal using either the test-to-control ratio (Fig. S4B) or the slope output (Fig. S4C). When these approaches are applied, very similar responses are obtained across the full concentration range despite the use of different serum samples. Furthermore, calibration curves constructed using the raw test line intensity (Fig. S4D) showed the greatest inter-assay error of ≤70%, whereas substantially lower inter-assay errors were obtained when using the ratio ≤20% or slope outputs ≤19% (Fig. S4E and F). The *R*^2^ value is also markedly improved following normalisation, with slope analysis providing a slight performance advantage over the ratio method. Interestingly, the limit of detection was not improved by applying the normalisation methods. We hypothesise that this is due to the shallow gradient of the test line intensity calibration curve, which is much lower compared to the ratio and slope methods which are almost the same, which limits sensitivity improvements. However, as the normalised outputs outperform the test line intensity across all other performance metrics, SERS-LFIA results should be normalised to enhance assay robustness and enable reliable comparison between different samples.

The primary objective of this study was to evaluate the long-term signal stability of the SERS-LFIA platform over a six-month period. However, first we assessed the reproducibility of the test by performing each sample in triplicate at all time points. To demonstrate the reproducibility, the SERS spectra obtained from the test line of all samples run on Day 0 are shown in Fig. S5. As expected, similar SERS signals were produced from each replicate. The RSD across the three samples using test line intensity of the 1230 cm^−1^ peak was calculated to give the of the test with 20%, 16% and 2% for the 0, 25 and 100 ng mL^−1^ samples being obtained respectively. The ratio and slope methods were then evaluated by calculating the RSD for triplicate measurements at each K18 concentration (0, 25, and 100 ng mL^−1^). When the T/C ratio was used, RSDs of 10%, 12%, and 4% were observed, providing an improvement in reproducibility for the 0 and 25 ng mL^−1^ samples compared to using test line intensity alone. Further improvement was achieved by calculating the slope across replicates, which yielded RSD values of 8%, 13%, and 2% for the 0, 25, and 100 ng mL^−1^ samples, respectively. These values were consistently lower than those obtained from either the test line intensity or ratio-based approach, demonstrating the enhanced reproducibility and precision of the regression slope method. All calculated RSD values across the three analytical approaches remained below 15%, indicating acceptable assay reproducibility according to common analytical standards. These results provide confidence that the SERS-LFIA generates consistent and quantitative outputs when freshly prepared. This reproducibility analysis was performed for each sample at all time points throughout the six-month stability study, with the corresponding RSD values summarised in Table 1.

**Table 1:** Summary of the six-month stability study of SERS-LFIA performance using three analytical approaches: peak intensity at the test line, T/C ratio, and regression slope of test *versus* control signal. For each time point and K18 concentration (0, 25, and 100 ng mL^−1^), the average signal and relative standard deviation (RSD) were calculated from triplicate measurements

Conc. of K18 (ng mL^−1^)	Time point	Average peak intensity	RSD (%)	Average ratio (T/C)	RSD (%)	Average slope (T *vs*. C)	RSD (%)
0	Day 0	822	20	0.03	10	0.05	8
	2 weeks	700	11	0.03	11	0.05	5
	1 month	701	24	0.03	24	0.05	10
	2 month	753	24	0.03	25	0.05	8
	3 month	573	6	0.02	15	0.04	21
	5 month	665	35	0.02	19	0.04	5
	6 month	659	13	0.03	40	0.05	18
25	Day 0	2098	16	0.07	12	0.09	13
	2 weeks	2863	14	0.10	1	0.11	4
	1 month	1686	17	0.08	18	0.09	17
	2 month	2246	7	0.08	15	0.01	9
	3 month	1456	10	0.06	10	0.08	6
	5 month	2679	17	0.09	2	0.11	3
	6 month	2628	10	0.10	21	0.11	14
100	Day 0	4559	2	0.17	4	0.18	2
	2 weeks	2979	10	0.15	14	0.17	7
	1 month	3636	10	0.15	15	0.17	7
	2 month	5208	3	0.2	6	0.21	3
	3 month	3646	29	0.13	18	0.16	12
	5 month	4114	16	0.16	1	0.16	10
	6 month	4183	13	0.2	6	0.23	4

When the test line SERS intensity was used as the primary output, the average RSD across all time points and concentrations was calculated to be 14.6%. However, considerable variability was observed between individual measurements. For example, an RSD of 3% was recorded for the 100 ng mL^−1^ sample after two months of storage, whereas this increased substantially to 29% after three months. Such high RSD values may indicate reduced reproducibility of the assay at certain time points.

Fortunately, normalising the SERS signal using the T/C ratio and the slope significantly improved the reproducibility of the assay output. Across all samples and time points, the average RSD values for the ratio and slope methods were reduced to 13.6% and 8.9%, respectively. For example, the 100 ng mL^−1^ sample measured at the three-month time point exhibited an RSD of 29% using raw test line intensity, decreased to 18% and 12% when using the ratio and slope methods, respectively. With the exception of the 0 ng mL^−1^ samples, most RSD values obtained *via* the ratio approach were below 20%. The higher RSD values observed for the blank (0 ng mL^−1^) samples are likely due to the inherently low SERS signal in the absence of K18, making the measurements more susceptible to small fluctuations in signal acquisition or background noise. This increased sensitivity to variation at low signal intensities is reflected in the higher relative error. The RSD values derived from the slope method were all below 20%, with the exception of a single data point that yielded an RSD of 21%. Overall, the slope-based output demonstrated the highest level of reproducibility among the three analytical approaches evaluated. These findings suggest that linear regression of the test *versus* control line signal offers a robust means of normalising SERS-LFIA results and should be considered a preferred method for future LFIA tests.

Next, we evaluated the change in SERS-LFIA output over the course of the six-month study to assess the performance of the test over time. This gave us information on the inter-assay precision. The degree of change was first calculated using the standard deviation of the output at each concentration across the six-month study. When the test line intensity was used as the measurement parameter, similar values were observed across all concentrations on both Day 0 and at 6 months, indicating that the performance of the test remained stable over time. The RSD values, across all time points, were calculated as 9%, 21%, and 17% for the 0, 25, and 100 ng mL^−1^ concentrations, respectively. While these values are generally acceptable, they do indicate some variability in the test performance, particularly at higher concentrations.

This variation was most notable in the 100 ng mL^−1^ sample, where the signal intensity was 2979 counts at week 2, compared to 5208 counts at month 2. This represents a significant discrepancy, with week 2 showing only 57% of the intensity recorded at month 2. Such variation limits the quantitative reliability of SERS-LFIAs. To mitigate this issue, the ratio method was employed. Using this approach, the signal at week 2 was 75% of that at month 2, which further improved to 80% when the slope method was applied.

However, the variability may not solely reflect assay performance but could instead arise from inherent inconsistencies in the positioning of the test line on the strip, which result from manual measurement and lab-based manufacturing processes. We estimate that laser misalignment of approximately ±0.5 mm relative to the point of maximum test line intensity can produce measurable changes in SERS signal. Therefore, the observed variation is more likely attributable to positional sensitivity than to NP stability or intrinsic test performance. To mitigate this issue, manual positioning can be improved by acquiring multiple measurements of the test and control lines to ensure that the maximum signal is captured. The cassette inserts used to maintain consistent alignment can also be optimised to achieve tighter tolerances. Furthermore, improved alignment guides to minimise user-dependent variability will be crucial for enhancing overall reproducibility. Additionally, large scale industrial manufacturing would mitigate inconsistences between tests. Automated fabrication processes allow for tighter control over critical parameters such as membrane cutting, reagent deposition and cassette assembly, which are often sources of variability. Techniques such as automated striping of capture reagents, precision lamination of membranes and robotic assembly can also ensure uniform placement and volume of reagents across batches, ultimately improving the reliability and reproducibility of signal measurements.

Importantly, the SERS signal at later time points returned to levels comparable to those seen on Day 0, which supports the conclusion that the test remained stable over time and that the observed variability was not due to degradation of the assay. The degree of change for the 0, 25, and 100 ng mL^−1^ samples using the ratio method were 17%, 16%, and 14%, respectively, and were further reduced to 10%, 13%, and 13% when the slope method was applied.

The inter-assay precision was evaluated for test line intensity, T/C ratio and slope response variables at each concentration and then combined in accordance with FDA Bioanalytical Method Validation Guidance for ligand-binding assays. The results are shown in Table S2. For 25 and 100 ng mL^−1^ samples, the inter-assay precision and the maximum and average values were generally ≤20% RSD across the six-month period, demonstrating acceptable reproducibility and assay stability. Higher variability was observed in the 0 ng mL^−1^ blank as expected and was acceptable for the lowest limit of quantification samples. Collectively, these results confirm acceptable assay precision and long-term stability, with the slope response producing the best inter-assay performance.^25^

**Fig. 3:**
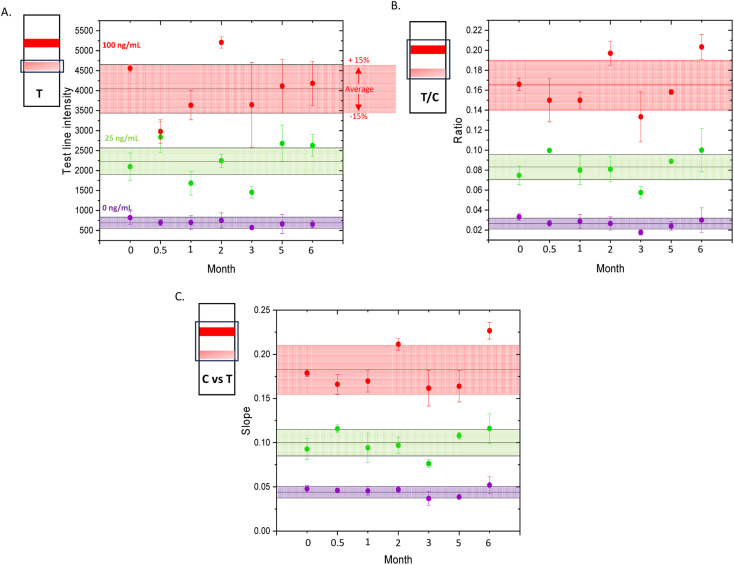
Quantitative output for each method, A. test line intensity, B. test/control ratio and C. control *vs*. test slope, showing the average value and standard deviation from three replicates per concentration (purple-0 ng mL^−1^, green-25 ng mL^−1^ and red-100 ng mL^−1^) measured at time points, day 0, 2 weeks, 1, 2, 3, 5, and 6 months. ±15% of the average value is highlighted.

To illustrate the signal variation over time and assess the reproducibility of each output method, scatter plots in Fig. 3 display the average and standard deviation for each output across three replicates per concentration at each time point. The overall average across all time points is indicated, along with a ±15% range, to highlight whether individual measurements fall within the predefined acceptable range. ±15% range was selected to be in line with FDA.

Fig. 3 A clearly shows that the test line intensity method yields the least reproducible results, with particularly large error bars observed for the 100 ng mL^−1^ samples after three months. While most average values remain within ±15% of the overall mean, this method exhibits greater variability over the six-month study period compared to the other output approaches. In contrast, the T/C ratio method produces smaller errors, with more average points falling within the ±15% range. As expected, the slope method demonstrates the best performance, with the least variation and highest reproducibility observed across replicates and throughout the time course of the study. It is worth noting that, for both the ratio and slope methods, the SERS-LFIA run at six months produced the highest values across all three concentrations. For the 100 ng mL^−1^ samples, the value exceeded the ±15% range. Initially, this anomaly was suspected to be due to a mis-spiking with a higher concentration. However, as a corresponding increase was not observed in the test line intensity method, this explanation was ruled out. Instead, it could be that the elevated signal is artificial, potentially caused by a weakened control line signal due to antibody degradation on the strip, which could affect normalisation and inflate the calculated values. It is possible for the test and control antibodies to degrade at different rates, as they originate from different species, can interact differently with the membrane, or have slightly different formulations, for example the control antibody was diluted in lab-prepared PBS buffer whereas the test line antibody wasn't. Future strategies to mitigate this and improve shelf life include the use of stabilisers such as BSA, trehalose, sucrose, or polyethylene glycol, as well as surface treatments to enhance protein binding and reduce nitrocellulose stress. Implementing these approaches can help synchronise the degradation rates of the test and control antibodies, maintaining consistent performance for beyond six months.

Based on the results, we have therefore determined that the current version of the SERS-LFIA strips remains stable for up to five months. When examining the slope outputs over time, the 0 ng mL^−1^ response decreased from 0.05 on Day 0 to 0.04 at five months, representing a 20% decrease. The 25 ng mL^−1^ response increased from 0.09 to 0.10, reflecting a 10% increase. Meanwhile, the 100 ng mL^−1^ response decreased from 0.18 to 0.16, corresponding to an approximate 11% decrease. This change in signal is generally acceptable as the test still meets its performance criteria of detecting K18 in serum and can clearly differentiate between the 3 concentrations at this time point. Furthermore, this result demonstrates that after 5 months stored at room temperature the test is still capable of discriminating a concentration of K18 that is representative of liver injury (100 ng mL^−1^) from a K18 value that does not represent liver injury (25 ng mL^−1^), indicating its value as a quantitative test for liver injury that can be used at the POC.

These findings underscore the importance of normalising SERS-LFIA outputs to account for variability in assay performance across different test runs. As previously discussed, such variability may arise from multiple sources, including NP flow inconsistencies, which could be attributed to NP instability over time. Additionally, mechanical misalignments, particularly those caused by variable pressure points within the 3D-printed cassette housing, can influence capillary flow dynamics. These issues are more likely to occur due to the inherent variability in lab-scale fabrication of both the SERS-LFIA strips and their cassettes. However, because NP flow impacts both the test and control lines simultaneously, normalising the test signal using either the test/control ratio or regression slope effectively compensates for these inter-device discrepancies. Thus, implementing such normalisation strategies is critical for ensuring reliable, reproducible, and analytically robust SERS-LFIA performance in real-world setting.

## Conclusion

We have demonstrated that silica-protected gold nanoparticles, combined with the slope derived linear regression analysis output enabled stable performance of SERS-LFIAs over a 5 month period. While this study confirms stability within that timeframe, future investigations will evaluate performance over longer durations, including up to one year. Good reproducibility was also achieved in triplicate human serum samples, with the normalisation effectively minimising variability due to serum protein binding, NP flow inconsistencies, and potential reductions in SERS signal over time. Importantly, the SERS-LFIA devices did not require refrigerated storage. They were stored in foil pouches with desiccant (silica), supporting their practicality for POC applications where cold storage may be unavailable or space is limited. This characteristic is particularly advantageous for use in resource-limited settings or decentralised clinics, ensuring reliable test performance under non-ideal storage conditions. Moreover, the platform's suitability for POC deployment was demonstrated by successfully analysing the SERS signals of both test and control lines using a HRR designed for field use. Overall, our results show that SERS-LFIAs offer a reliable, reproducible, and sensitive diagnostic solution over five-months, supporting their utility in real-world clinical and decentralised healthcare settings.

## Author contributions

S. S. D., B. C., K. M. S., F. S. and J. F. performed the experiments and analysed the data. P. F., J. M. and S. L. project managed the programme of work. C. R., D. C., and D. B., developed the handheld readers. C. W., J. W. D., K. F. and D. G. supervised the project.

## Conflicts of interest

C. R., D. C., D. B., J. F. and M. Z. are employees of Wasatch Photonics. S. S. D., B. C., K. M. S., J. W. D., K. F. and D. G. have filed a patent application related to the LFIA (Patent Application No. GB2314603.8, filed 22 September 2023). This technology relates to the detection methods described in the current study. The remaining authors declare no competing interests.

## Ethical statement

All experiments were performed in accordance with the University of Strathclyde's ethical framework which are aligned to the Declaration of Helsinki and approved by the ethics committee at the University of Edinburgh and the University of Strathclyde. Informed consents were obtained from human participants of this study.

## Supplementary Material

AN-151-D6AN00053C-s001

## Data Availability

The data used in this study are available on the Pure portal (https://doi.org/10.15129/d16388a9-4a87-4c99-a6e8-060859a25416). Supplementary information (SI) is available. See DOI: https://doi.org/10.1039/d6an00053c.
