# Supplementary material for: Development of a surface enhanced Raman scattering lateral flow immunoassay with prolonged reproducibility and stability over time
Source: Analyst. 2026 Apr 2;151(11):3130–40. doi: 10.1039/d6an00053c (PMC13088774; doi:10.1039/d6an00053c)
Supplement: AN-151-D6AN00053C-s001 [file AN-151-D6AN00053C-s001.pdf]

Supplementary Information (SI) for Analyst.
This journal is © The Royal Society of Chemistry 2026

Supplementary information

Development of a surface enhanced Raman scattering lateral flow immunoassay with prolonged reproducibility and stability over time

Sian Sloan-Dennison[1], Ben Clark[1], Kathleen M. Scullion[2], Fiona Smillie[1], Stacey Laing[1], Paul Fineran[2], Joanne Mair[2], Cicely Rathmell[3], Dieter Bingeman[3], Jonathan Faircloth[3], David Creasey[3], Neil Shand[4], Chris J. Weir[5], James W. Dear[2], Duncan Graham[1], Karen Faulds[1]

1.Department of Pure and Applied Chemistry, Technology and Innovation Centre, 99 George Street, Glasgow, UK

2. Centre for Cardiovascular Science, The Queen's Medical Research Institute, University of Edinburgh, Edinburgh, UK

3. Wasatch Photonics, Morrisville, NC, USA

4. Defence Science and Technology Laboratory, Porton Down, Salisbury, UK

5. Edinburgh Clinical Trials Unit, Usher Institute, University of Edinburgh, Edinburgh, UK

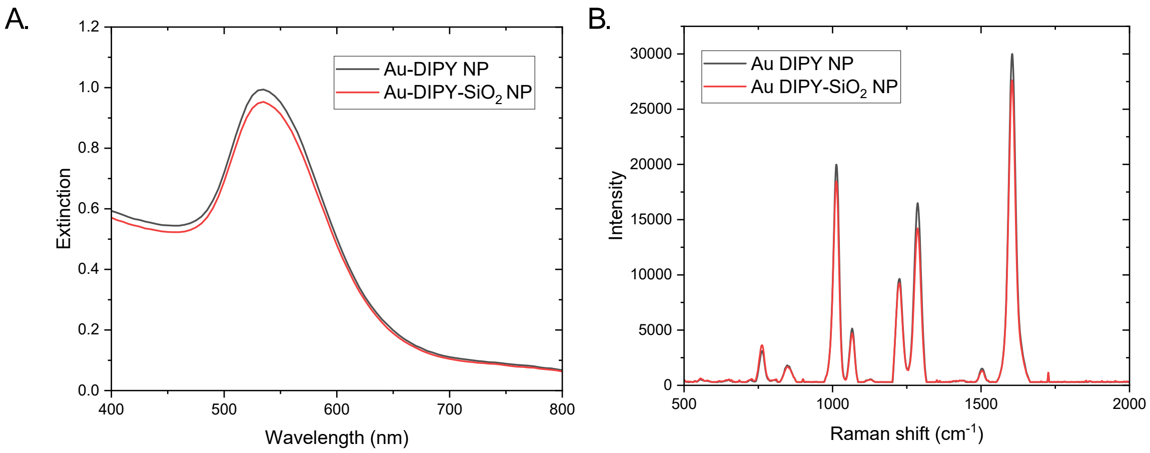

Figure S1. Characterisation of silica shell A. Extinction spectra and B, SERS spectra of Au-DIPY-NP (black) and Au-DIPY-SiO2-Ab NP (red). SERS spectra collected with a 785 nm laser excitation, 40 mW laser power, 1 second acquisition.

Table S1 Size and zeta potential of gold nanoparticles (Au NP) and gold nanoparticles encapsulated in silica (Au-SiO_2 NP) when incubated with 5, 10 and 15 µL of serum. The values are the average and standard deviation of three measurements.

Sample	Size (nm)	Zeta potential (mV)
Au NP	56 ±2	-34 ± 1
Au NP +5 µL of serum	1836 ± 287	0.1 ± 0.2
Au NP + 10 µL of serum	2046 ± 473	62 ± 25
Au NP + 15 µL of serum	2869 ± 309	21 ± 20
Au-SiO_2 NP	68 ± 1	-24 + 1
Au-SiO_2 NP +5 µL of serum	90 ± 4	-13.3 ± 1
Au-SiO_2 NP +10 µL of serum	92 ± 4	-10 ± 1
Au-SiO_2 NP +15 µL of serum	101 ± 8	12 ± 1

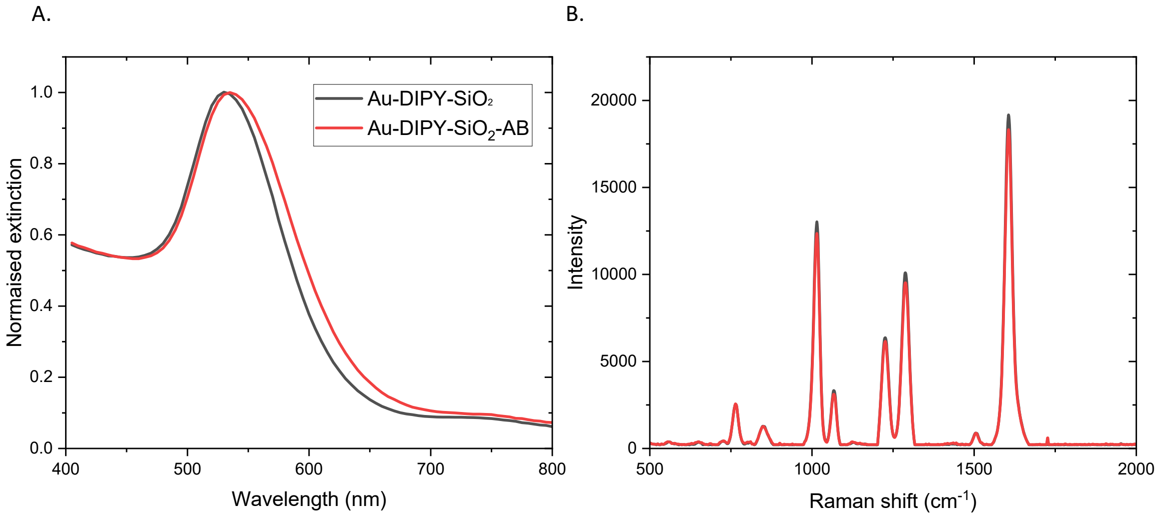

Figure S2 Characterisation of conjugates A. Normalised extinction spectra and B, SERS spectra of Au-DIPY-SiO_2 NP (black) and Au-DIPY-SiO2-Ab NP (red). SERS spectra collected with a 785 nm laser excitation, 40 mW laser power, 1 second acquisition.

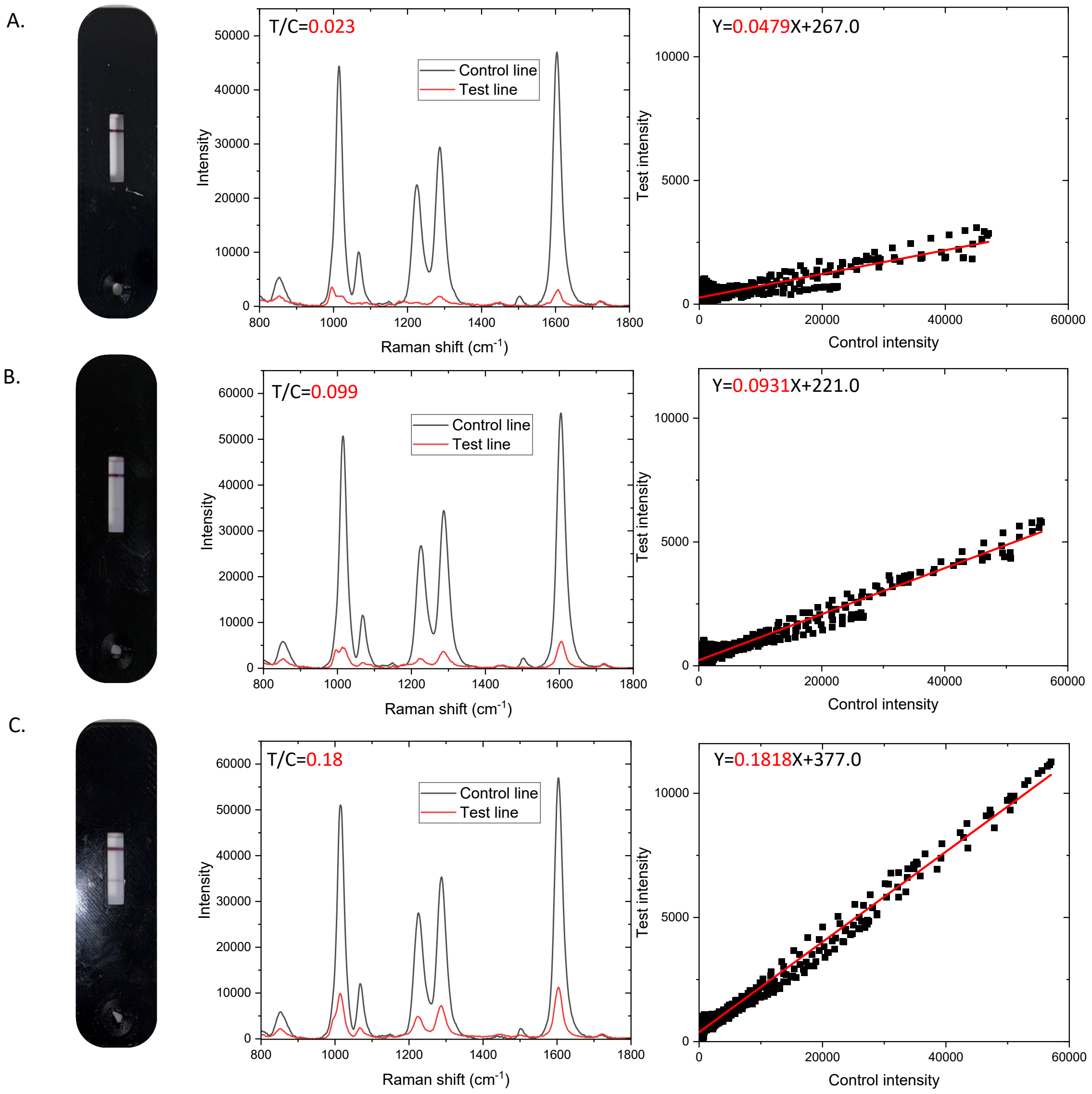

Figure S3 Results of SERS-LFIA run with serum spiked with A. 0 ng/mL, B. 25 ng/mL and c. 100 ng/mL of K18 on Day 0. The figure shows an image of the SERS-LFIA, the SERS spectra taken from control line (black) and test line (red) with T/C ratio and the linear regression analysis of control vs test intensity and associated equation of line with the gradient (slope) output shown in red The SERS signal of the test and control lines were measured using a Wasatch Photonics HRR with 785 nm laser excitation, 3.6 mW laser power and 1 second acquisition.

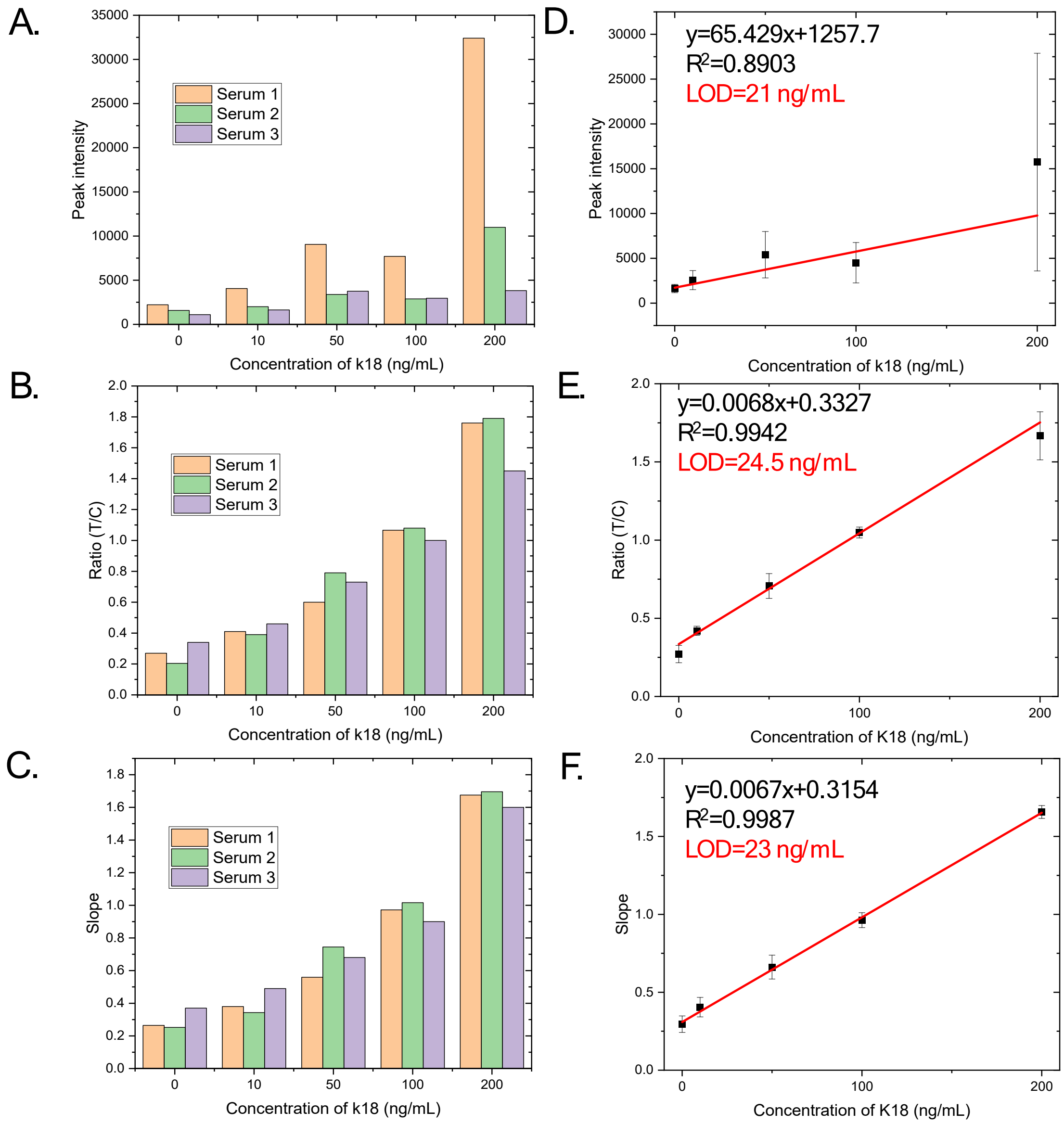

Figure S4. Bar chart showing the results of a triplicate calibration study performed using sera 1, 2, and 3. Comparison of A. test line intensity, B. ratio (T/C), and C. slope output. Calibration curves of spiked K18 concentration plotted against D. test line intensity, E. ratio (T/C), and F. slope. Data points represent the average ± standard deviation obtained from the three sera.

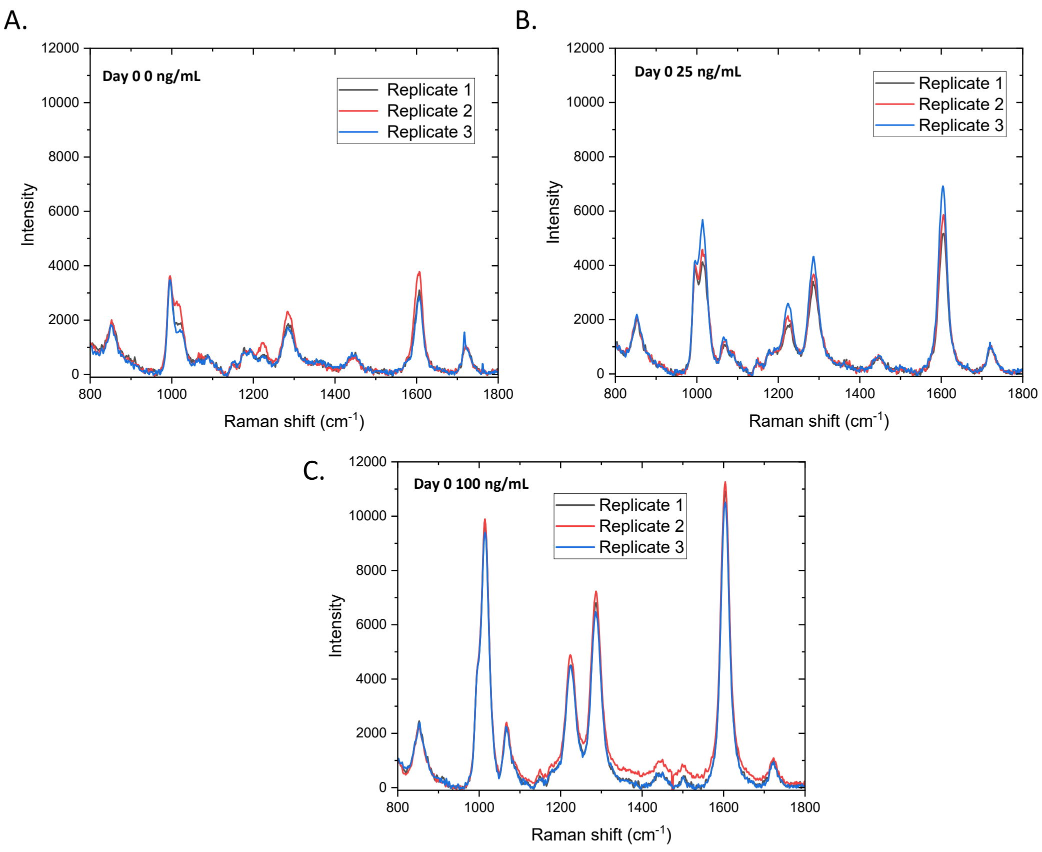

Figure S5. SERS spectra of test line from SERS -LFIA run on Day 0 with serum samples spiked with A. 0 ng/mL, B. 25 ng/mL C. 100 ng/mL. Each sample was run in triplicate. The SERS signal of the test lines were measured using a Wasatch Photonics HRR with 785 nm laser excitation, 3.6 mW laser power and 1 second acquisition

Table S2 Maximum and average inter-assay performance values for each method

Analysis	0 ng/mL	25 ng/m L	100 ng/m L	Overall inter-assay performance
Peak intensity	≤ 35 % (19 %)	≤20 (13 %)	Mostly below ≤20 % (12 %)	15 %
Ratio	≤40% (20 %)	Mostly below ≤20 % (11 %)	≤18 % (9 %)	14 %
Slope	≤21 % (11 %)	≤17 % (9 %)	≤12 % (6 %)	9 %
FDA acceptance	Acceptable	Pass	Pass	Pass
